# Response to: Comment on: “Effects of Plyometric Training on Physical Performance: An Umbrella Review”

**DOI:** 10.1186/s40798-023-00609-0

**Published:** 2023-08-15

**Authors:** Rafael L. Kons, Lucas B. R. Orssatto, Jonathan Ache-Dias, Kevin De Pauw, Romain Meeusen, Gabriel S. Trajano, Juliano Dal Pupo, Daniele Detanico

**Affiliations:** 1 Department of Education, Faculty of Education, Federal University of Bahia, Salvador, Bahia 40110-100 Brazil; 2 School of Exercise and Nutrition Sciences, Faculty of Health, Queensland University of Technology, Brisbane, QLD Australia; 3 Research Group On Technology, Sport and Rehabilitation, Catarinense Federal Institute - IFC, Araquari, Brazil; 4 Human Physiology and Sports Physiotherapy Research Group and Brussels Human Robotic Research Center (BruBotics), Vrije Universiteit Brussel, Pleinlaan 2, 1050 Brussels, Belgium; 5 Biomechanics Laboratory, Centre of Sports - CDS, Federal University of Santa Catarina, Florianópolis, Santa Catarina Brazil

Dear Editor,

We agree that letters to the editor are part of the scientific process, and, in fact, we aim to clarify the methodological concerns about our manuscript. Notwithstanding, we understand the minor concerns expressed in the letter and will discuss each aspect and make the necessary amendments, as follows.

## Literature Search Strategy

In our umbrella review, we selected 29 studies based on a priori defined inclusion/exclusion criteria (see Kons et al. [1]—Table 1). Ramirez-Campillo et al. [2] questioned why we did not include in our review a list outlining the reasons why 47 studies were excluded. However, this information is clearly listed in Fig. 1 of the manuscript, as follows: “Reports excluded: No meta-analysis included related to the plyometric training (*n* = 17); Reviews related to combined training (*n* = 10); Reviews related to post-activation potentiation (*n* = 2); Reviews related to lower limb injury prevention (*n* = 6); Reviews related to the influence of stretching on the lower limbs (*n* = 2); Reviews involving complex training (*n* = 10)”.

Ramirez-Campillo et al. [2] also listed a few meta-analyses excluded from our study that took into account other comparators, such as strength training [3–5]. However, it is important to clarify that comparing plyometric training to other interventions was not part of our study aims, as follows: “(i) to systematically review the available meta-analytical evidence that has examined the effects of plyometric training on physical fitness performance (e.g., sprint time, change of direction, maximal strength, muscle power and explosive strength, vertical or horizontal jump and specifying additional outcomes, such as endurance, high intermittent running performance, kicking performance, balance, and Yo-Yo intermittent recovery test) in different populations”.

The eight meta-analyses mentioned by Ramirez-Campillo et al. [2] as not being included in our review were published after the conclusion of the systematic search. It is great that the field is moving forward very quickly, and an update to our review might be necessary in the next 3–5 years, hopefully including higher-quality meta-analyses (e.g., including randomized controlled trials [RCTs] only).

## Interpretation of Published Meta-analyses

The eligibility criteria for our umbrella review (see Table 1—Kons et al. [1]) included studies with a control group or control situation. However, when developing the present study, we noted that only a few meta-analyses actually compared intervention to control groups. Therefore, we chose to highlight that most of the selected studies lacked comparisons with control groups, and they were classified as low-to-moderate quality. We also selected and discussed studies with comparisons between groups (e.g., vertical vs. horizontal jumps), but we considered as a central point a direct comparison of the effects of plyometric training in relation to controls. By using a control group, researchers can ensure that any observed differences in outcomes between the experimental and control groups are a result of the intervention or treatment being tested, rather than other extraneous factors. This helps to minimize bias and increases the validity of the study's findings. Control groups in RCTs are essential as they provide a basis for comparison and enable researchers to determine the effectiveness of new treatments or interventions being tested by establishing a baseline and minimizing bias. Despite this, we understand the importance of the benefits of plyometric training when establishing comparisons with other types of intervention (e.g., horizontal training, running, and others), and this could represent a great opportunity for investigation in the future. The correction related to the data is present in an additional file 1.

## Inconsistent and Erroneous Data

The minor errors and respective corrections are described in the Additional file 1. Regarding the authors’ [2] concern that “it is unclear how standardized mean differences [SMDs] were computed from meta-analyses that reported different types of effect sizes (e.g., Hedges’ g; standardized mean differences)”, we understand that different types of SMD calculations used in meta-analysis can introduce bias into the generated effect size [6]. Unfortunately, the literature is inconsistent regarding the methods used for synthesizing SMDs, and accurately converting effect sizes from the available data is not possible. However, minimal differences have been reported when synthesiing SMDs and 95% confidence intervals by different methods in meta-analyses [6]. Therefore, we disagree that the inconsistency of how SMDs were computed in the meta-analyses would have influenced the interpretation of the data.

We appreciate the authors’ [2] contributions to improving our comprehensive review. As the years go by and there is continued growth in the use of plyometric training applications in sport, rehabilitation and across the lifespan, in our opinion, there will always be room for further analyses, particularly considering original studies (e.g., RCTs) and other comprehensive reviews.

### Supplementary Information

**Additioanl file 1:** Correction “Effects of Plyometric Training on Physical Performance: An Umbrella Review”.

## Data Availability

Not applicable.
