# Supplementary material for: Response to: Comment on: “Effects of Plyometric Training on Physical Performance: An Umbrella Review”
Source: Sports Med Open. 2023 Aug 15;9:74. doi: 10.1186/s40798-023-00609-0 (PMC10425302; doi:10.1186/s40798-023-00609-0)
Supplement: Supplementary file 1 — Additioanl file 1: Correction “Effects of Plyometric Training on Physical Performance: An Umbrella Review”. [file 40798_2023_609_MOESM1_ESM.docx]

**Correction “Effects of Plyometric Training on Physical Performance: An Umbrella Review”**

**Table 2.**

Berton et al. 2018 [1]. Correction N = 76

Moran et al. 2018 [2] Correction N = 199

Moran et al. Correction = 0.66 (0.33–0.98, p = 0.0001)

Asadi et al. 2016 [11]. Correction to youth and adult practitioners and non-practitioners of sports.

Ramirez-Campillo et al. 2020 [4] change effect size of “2.07” for 0.82

Berton et al. 2018 [1] change I^2^ = 21% to I^2^ = 0% reported in the original meta-analysis.

Ramirez-Campillo et al. 2020 [5] Correction; the Statistical model “Within-subject SMD (Hedges’ g)”

**References**

[1] Berton R, Lixandrão ME, Pinto ESCM, Tricoli V. Effects of weightlifting exercise, traditional resistance and plyometric training on countermovement jump performance: A meta-analysis. J Sports Sci. 2018;36(18):2038-44

[2] Moran J, Ramirez-Campillo R, Granacher U. Effects of jumping exercise on muscular power in older adults: A meta-analysis. Sports Med. 2018;48(12):2843-57

[3] Asadi A, Arazi H, Young WB, Saez de Villarreal E. The effects of plyometric training on change-of-direction ability: A meta-analysis. Int J Sports Physiol Perform. 2016;11(5):563-73

[4] Ramirez-Campillo R, Andrade DC, Nikolaidis PT, Moran J, Clemente FM, Chaabene H, et al. Effects of plyometric jump training on vertical jump height of volleyball players: A systematic review with meta-analysis of randomized-controlled trial. J Sports Sci Med. 2020;19:489-99

[5] Ramirez-Campillo R, Hermoso AG, Moran J, Chaabene H, Negra Y, Scanlan AT. The effects of plyometric jump training on physical fitness attributes in basketball players: a meta-analysis. J Sport Health Sci. 2020;24:S2095-2546(20)30169-1.
